# Identifying Patient Characteristics Associated With Opioid Use to Inform Surgical Pain Management

**DOI:** 10.1097/AS9.0000000000000355

**Published:** 2023-11-14

**Authors:** Anish K. Agarwal, Ruiying Xiong, Jeffrey Ebert, Fran Shofer, Evan Spencer, Daniel Lee, Zarina Ali, M. Kit Delgado

**Affiliations:** From the * Department of Emergency Medicine, University of Pennsylvania, Philadelphia, PA; † Leonard Davis Institute of Health Economics, University of Pennsylvania, Philadelphia, PA; ‡ Penn Medicine Center for Health Care Innovation, University of Pennsylvania, Philadelphia, PA; § Department of Surgery, Division of Urology, University of Pennsylvania, Philadelphia, PA; ‖ Department of Neurosurgery, University of Pennsylvania, Philadelphia, PA.

**Keywords:** learning health systems, mobile health, opioid prescribing, quality improvement

## Abstract

**Objective:**

Balancing surgical pain management and opioid stewardship is complex. Identifying patient-level variables associated with low or no use can inform tailored prescribing.

**Methods:**

A prospective, observational study investigating surgical procedures, prescription data, and patient-reported outcomes at an academic health system in Pennsylvania. Surgical patients were consented following surgery, and prospective data were captured using automated text messaging (May 1, 2021–February 29, 2022). The primary outcome was opioid use.

**Results:**

Three thousand six hundred three (30.2%) patients consented. Variation in patient reported used included 28.1% of men reported zero use versus 24.3% of women, 20.5% of Black patients reported zero use versus 27.2% of white patients. Opioid-naïve patients reported more zero use as compared with chronic use (29.7% vs 9.8%). Patients reporting higher use had more telephone calls and office visits within 30 days but no change in emergency department utilization or admissions. Higher discharge pain score was associated with higher use. In the adjusted analysis of opioid use relative to the guideline, higher use was associated with age, male sex, obesity, discharge pain score, and history of mental health disorder. In the adjusted model, younger age and being opioid naïve were associated with low to zero use across procedures.

**Conclusions:**

Younger age, being opioid-naïve, and lower discharge pain score are associated with low or no postoperative opioid use. These characteristics can be used by clinicians to help tailor opioid prescribing to specific patients to reduce the risk of prolonged exposure and unused tablets in the community.

## Abstract 2

Mini-Abstract

*Question:* Are there identifiable patient-level characteristics associated with low or no opioid use following common surgical procedures.

*Findings:* In this observational cohort study of 3603 surgical patients, 32.6% report no or low use of their opioid prescription. Discharge pain score, being opioid-naïve, and younger age were associated with no or low use in the adjusted analyses.

*Meaning:* Patient-reported outcomes data reveal opportunities to tailor postoperative opioid prescribing using easily identifiable patient characteristics (age and previous opioid exposure) and routinely collected discharge pain score.

Managing postoperative pain remains a complex challenge within the backdrop of the opioid epidemic.^1–3^ Surgeons must weigh the potent analgesia offered by opioids against the risks of adverse effects, the potential for long-term use, and misuse.^2,4,5^ Surgical research has demonstrated a persistent mismatch between the number of opioid tablets prescribed and the number of tablets patients use.^6–8^ This mismatch and excess can result in unused opioid tablets left in homes, which pose a direct risk to patients, their families, and members of the community.^9,10^ The practice of opioid stewardship, akin to antibiotic stewardship, attempts to adequately address pain, while minimizing the duration and quantity of acute opioid prescriptions.^11^ Accreditation bodies, surgical societies, state committees, and local institutions have developed pain management practices, and some include procedure-specific guidelines for prescribers.^12–14^

While there have been advances in the development and implementation of procedure specific prescribing guidelines to promote opioid stewardship,^6,8,15–17^ most guidelines recommend a single default number of tablets meant to cover a majority of patients’ needs. Research in gynecologic surgery reveals associations with higher opioid use across a variety of patient characteristics (eg, chronic pain, exposure to opioids, depression, and anxiety) and expectations of anticipated postoperative pain.^18^ Similar research in hepatic surgery noted higher opioid use among younger patients and those with history of pain medication usage prior to surgery.^19^

What remains unknown is whether there are identifiable patient-level characteristics that predict opioid use following surgery and if these generalize across procedure types. More data are needed to determine which patient characteristics or variables can be used to support a more tailored approach to opioid prescribing, adequately address pain, and minimize prolonged exposure and excess. This evidence gap inhibits the development of actionable recommendations for prescribers to enable tailoring specific opioid amounts to patient characteristics across a breadth of procedures. Uncovering these insights would facilitate a precision medicine approach to pain management and opioid stewardship, which could include procedure-specific guidelines stratified by patient characteristics, shared decision-making tools, and electronic health record clinical decision support tailored to patient characteristics.

The objective of this study was to identify patient-level factors associated with opioid use following high volume surgical procedures using prospectively collected patient-reported outcomes data via an institutional postoperative automated text messaging system.

## METHODS

### Study Population and Period

This study was conducted within the broader quality improvement landscape at a health system focused on optimizing postoperative pain management and promoting opioid stewardship. As previously described, this health system developed and launched an automated text messaging system to prospectively collect patient-reported outcomes following surgery alongside electronic health record data.^11,17,20,21^

The study population consisted of patients 18 and older with a mobile phone number who were prescribed opioids for postoperative pain control after undergoing one of the 30 highest volume surgical procedures (Appendix) at the University of Pennsylvania Health System (“Penn Medicine”) between May 1, 2021, and February 29, 2022. Before the study period, Penn Medicine and this research team developed recommended opioid prescribing guidelines for surgical procedures. These guidelines were created by prior local quality improvement data collection and from existing external recommendations.^13,17^ These guidelines were developed and approved by all surgical department and division clinical representatives. The guidelines served as recommendations, were posted on health system websites, and circulated to clinicians but were not mandatory or required.

This study was approved by the institutional review board of the University of Pennsylvania. The study followed the Standards for Quality Improvement Reporting Excellence (SQUIRE) reporting guidelines.^22^

### Data Collection

Eligible patients were automatically enrolled in an automated text messaging program that prospectively collected patient-reported outcomes and opioid use in the 28 days following surgery as part of usual care. The automated text messaging prospectively collects patient-reported outcomes (eg, pain intensity and ability to manage pain) and prescription use. Participants received a series of text messages following discharge; the initial text message obtained their electronic written consent. Patients had the ability to opt out of text messaging at any time.

Consenting patients were asked: (1) whether they filled their opioid prescription; (2) their pain intensity on a scale of 0 to 10, with 0 being “no pain”; (3) their ability to manage pain on a scale of 0 to 10, with 0 being “not at all able”; and (4) their opioid use in number of tablets. Patients were then asked whether they planned to take opioids to manage pain in the upcoming days. Patients reporting planned future opioid use were queried on subsequent postoperative days 7, 14, 21, and 28. When a patient reported no use or no further use beyond day 7, the text messaging ended. Additional questions, not reported in this analysis, included opioid disposal and number of days it took for pain to resolve. Prescribers were not directed to change their standard of care when discussing pain management, use of analgesics, or prescribing practice. Patient information was deidentified and stored securely. Demographic information, comorbidities, surgical procedure, and discharge pain score were obtained from the electronic health record.

### Outcomes

The primary outcome was self-reported opioid use converted to oxycodone 5 mg tablet equivalents.^23^ Opioid use was categorized into low, medium, and high opioid use by terciles of (1) proportion of tablets used relative to prescription amount and (2) proportion of tablets used relative to guideline recommendation. We assessed this at the level of the surgical procedure. We chose this approach to assess heterogeneity in self-reported opioid use because the commonly used alternative of assessing amount taken versus amount prescribed may be subject to known biases and disparities in prescribing. For example, there is evidence that Black patients are prescribed less opioids than White patients for similar conditions.^24,25^ To assess the sensitivity of findings regarding heterogeneity in self-reported opioid use, we did analyze self-reported opioid use relative amount prescribed as a secondary outcome.

Other secondary outcomes included self-reported pain intensity, ability to manage pain, and proportion of opioid prescription used relative to total quantity prescribed and relative to the surgical procedure guideline. We also extracted secondary outcomes on health care use in 30 days following the procedure from the EHR including emergency department visits, telephone encounters, and outpatient visits. Surgical procedure guidelines were developed locally at this health system, informed by existing literature, and were in place before the study timeline.

Key independent variables abstracted from the medical record included patient-level demographics, surgical procedure, Elixhauser Comorbidity Index, history of mental health disorder, history of illicit substance use disorder, and prior opioid prescription history in the health system. Patients with no prior opioid prescriptions were categorized as “opioid naïve.” Patients with >90 days of opioids in the prior 180 days were categorized as being on chronic opioid therapy or “chronic use.” All other patients fitting between these definitions were categorized as being intermittently on opioids or labeled “intermittent use.”

### Statistical Analysis

All statistical analysis was performed using R Statistical Software (version 4.2.2). Chi-squared test and t-test were used to assess the baseline differences between text responders and nonresponders, and among patients with zero, low, medium, and high opioid consumption. We then compared differences in the primary and secondary outcomes according to tercile level of opioid use relative to quantity prescribed and guideline recommendation.

We then used a linear-mixed model with random effect of procedure group was used to estimate the association between the ratio of the self-reported opioid use relative to the guideline recommended amount and patient characteristics including age, gender, race, body mass index, pain score reported at discharge, Elixhauser score, illicit substance use disorder, mood disorder, psychotic disorder, alcohol use disorder, tobacco use disorder, and previous opioid use. We repeated these models for the outcome of self-reported opioid use to amount prescribed.

As post hoc analysis based on identifying being opioid-naïve and last pain score at discharge, we use procedure-specific models using the same covariates as above to estimate the predicted number of tablets taken using average marginal effects. Patient status as being opioid-naïve and last pain score (0–3, 4–6, 7–10) were entered in the model as interaction terms. We included the results from the 2 highest volume procedures in the main manuscript and report this for all 30 procedures in the supplement (http://links.lww.com/AOSO/A269).

## RESULTS

### Text Messaging and Response

During the study period, 11,920 patients were eligible to participate and were sent a text message invitation. A total of 3603 (30.2%) patients consented and completed questions. Compared with nonresponding patients, patients in the responding group were more likely to be male (48.3% vs 45.6%), younger age (mean age 55.2 years vs 57.3 years), white (79.0% vs 63.0%), opioid-naïve (77.3% vs 67%), and have fewer medical comorbidities (Table 1). Patients underwent 1 of 30 common surgical procedures with the most frequent being knee arthroplasty (n = 637, 17.7%).

**TABLE 1.** Respondents versus Nonrespondents

	Responder	Nonresponder	*P*
Participants (n)	3603	8317	-
Mean age (SD)	55.24 (15.18)	57.28 (15.56)	<0.001
Age range, n(%)			<0.001
18–34	433 (12.2)	867 (10.7)	
35–54	1050 (29.7)	2122 (26.2)	
55+	2054 (58.1)	5111 (63.1)	
Gender, n(%)			0.002
Female	1797 (49.9)	4307 (51.8)	
Male	1740 (48.3)	3793 (45.6)	
Not recorded	66 (1.8)	217 (2.6)	
Race, n (%)			<0.001
White	2846 (79.0)	5237 (63.0)	
Black	478 (13.3)	2150 (25.9)	
Other	279 (7.7)	930 (11.2)	
Body mass index, n(%)			0.001
<18.5 (underweight)	18 (0.5)	78 (0.9)	
18.5–24.9	817 (22.7)	1830 (22.0)	
25–29.9 (overweight)	1227 (34.1)	2675 (32.2)	
30 to 39.9 (obese)	1195 (33.2)	2776 (33.4)	
40 or more (morbidly obese)	344 (9.6)	957 (11.5)	
Opioid use n(%)*			
Opioid-naïve	2735 (77.3)	5425 (67.0)	<0.001
Intermittent opioid use	757 (21.4)	2456 (30.3)	<0.001
Chronic use	51 (1.4)	232 (2.9)	<0.001
Elixhauser index, n(%)**			<0.001
0	1335 (37.1)	2447 (29.4)	
1	889 (24.7)	1850 (22.2)	
2–3	984 (27.3)	2426 (29.2)	
4+	395 (11.0)	1594 (19.2)	
History of mood disorder, n (%)**	192 (5.3)	506 (6.1)	0.116

* Pain Score Scale 0–10, 10 being highest pain.

** Ability to Manage Pain Score Scale 0–10, 10 being the most able to manage pain.

### *Patient-reported Pain, Opioid Use, and Follow*-*up Encounters*

Low, medium, and high opioid use were categorized by terciles of proportion of prescription tablets used of total prescription quantity. Significant demographic differences existed whereby 28.1% of men reported zero use and 21.1% of women reported medium use. By race, 20.5% of Black patients versus 27.2% of white patients reported zero use, while 41.6% of Black patients and 30.7% of white patients reported high use. There was significantly higher opioid use among patients with more medical comorbidities, higher Elixhauser index, history of mood disorders, and history of substance use disorder. Opioid-naïve patients reported more zero use as compared with those with chronic use (29.7% vs 9.8%; Table 2).

**TABLE 2.** Patient-Reported Opioid Use and Patient-Level Variables

	Patient-Reported Opioid Use				
	Zero	Low	Medium	High	*P*
Participants (n)	940	834	682	1147	–
**Patient demographic data**					
Mean age (SD)	54.97 (16.01)	56.53 (15.20)	54.72 (14.90)	54.86 (14.57)	0.054
Age range, n(row %)					0.281
18–34	128 (29.6)	87 (20.1)	85 (19.6)	133 (30.7)	
35–54	266 (25.3)	228 (21.7)	206 (19.6)	350 (33.3)	
55+	532 (25.9)	497 (24.2)	381 (18.5)	644 (31.4)	
Gender, n (%)					0.006
Female	437 (24.3)	413 (23.0)	380 (21.1)	567 (31.6)	
Male	489 (28.1)	399 (22.9)	292 (16.8)	560 (32.2)	
Not recorded	14 (21.2)	22 (33.3)	10 (15.2)	20 (30.3)	
Race, n (%)					<0.001
White	773 (27.2)	657 (23.1)	543 (19.1)	873 (30.7)	
Black	98 (20.5)	91 (19.0)	90 (18.8)	199 (41.6)	
Other	69 (24.7)	86 (30.8)	49 (17.6)	75 (26.9)	
Body mass index, n(%)					<0.001
<18.5 (underweight)	265 (32.4)	187 (22.9)	141 (17.3)	224 (27.4)	
18.5–24.9 (normal weight)	7 (38.9)	5 (27.8)	2 (11.1)	4 (22.2)	
25–29.9 (overweight)	335 (27.3)	283 (23.1)	232 (18.9)	377 (30.7)	
30–39.9 (obese)	260 (21.8)	285 (23.8)	222 (18.6)	428 (35.8)	
40 or more (morbidly obese)	72 (20.9)	74 (21.5)	84 (24.4)	114 (33.1)	
Opioid use n(%)*					
Opioid-naïve	813 (29.7)	621 (22.7)	509 (18.6)	792 (29.0)	<0.001
Intermittent opioid use	110 (14.5)	182 (24.0)	155 (20.5)	310 (41.0)	<0.001
Chronic use	5 (9.8)	10 (19.6)	9 (17.6)	27 (52.9)	0.005
Elixhauser index, n(%)**					
0	374 (28.0)	318 (23.8)	246 (18.4)	397 (29.7)	
1	240 (27.0)	207 (23.3)	153 (17.2)	289 (32.5)	
2–3	234 (23.8)	219 (22.3)	200 (20.3)	331 (33.6)	
4+	92 (23.3)	90 (22.8)	83 (21.0)	130 (32.9)	
History of mood disorder, n (%)**	33 (17.2)	42 (21.9)	34 (17.7)	83 (43.2)	0.002

* Pain Score Scale 0–10, 10 being highest pain.

** Ability to Manage Pain Score Scale 0–10, 10 being the most able to manage pain.

Higher discharge pain score was associated with higher opioid use, as was higher day 7 pain intensity. Conversely, higher patient reported ability to manage pain was reported with lower overall opioid use. Patients reporting higher opioid use also had a significantly greater number of telephone encounters and office visits with their surgical provider within 30 days but no change in emergency department utilization or hospital admissions. Patients with higher opioid use also had significantly more opioid prescription refills within 30 days of surgery (Table 3). Patient-level characteristics associated with higher use *relative to prescription quantity* included older patients, male patients, Black race, history of substance or alcohol use disorder, and higher discharge pain score (Supplement http://links.lww.com/AOSO/A269). Within the total cohort, 940 patients (26.1%) reported no opioid use, 1582 (44.6%) were opioid-naïve and had a low discharge pain score, and 334 (9.4%) where less than 40 years of age, opioid-naïve, and had a low discharge pain score (Supplement http://links.lww.com/AOSO/A269).

**TABLE 3.** Patient-Reported Outcomes and Follow Up

	Patient-Reported Opioid Use				
	Zero	Low	Medium	High	*P*
Participants (n)	940	834	682	1147	-
Last pain score	2.11 (2.24)	3.06 (2.47)	3.47 (2.53)	4.09 (2.57)	<0.001
Severity of pain scores, n (%)					<0.001
Mild (0–3)	683 (34.8)	481 (24.5)	350 (17.8)	448 (22.8)	
Moderate (4–6)	206 (18.0)	260 (22.7)	228 (19.9)	449 (39.3)	
Severe (>6)	43 (9.1)	85 (18.0)	103 (21.8)	242 (51.2)	
Pain score at 7 days, mean (SD)*	2.25 (1.88)	3.15 (2.04)	3.71 (2.14)	4.94 (2.29)	<0.001
Ability to manage pain, mean (SD)**	8.42 (2.61)	7.84 (2.79)	7.75 (2.49)	6.85 (2.46)	<0.001
**Postsurgery encounters and prescriptions, mean (SD**)					
Office visits within 30 days	1.09 (1.01)	0.97 (1.00)	1.08 (0.99)	1.05 (0.95)	0.048
Telephone encounters within 30 days	1.73 (2.40)	1.87 (2.69)	2.19 (2.48)	2.50 (2.78)	<0.001
Hospital admissions within 30 days	0.02 (0.13)	0.02 (0.14)	0.01 (0.13)	0.03 (0.19)	0.228
ED visits within 30 days	0.03 (0.18)	0.05 (0.24)	0.04 (0.21)	0.05 (0.29)	0.133
Opioid refills within 30 days	1.23 (0.57)	1.08 (0.28)	1.32 (0.64)	1.71 (1.08)	0.005

* Pain Score Scale 0–10, 10 being highest pain.

** Ability to Manage Pain Score Scale 0–10, 10 being the most able to manage pain.

### Opioid Use Relative to Surgical Procedure Guideline Recommendations

**FIGURE 1.**
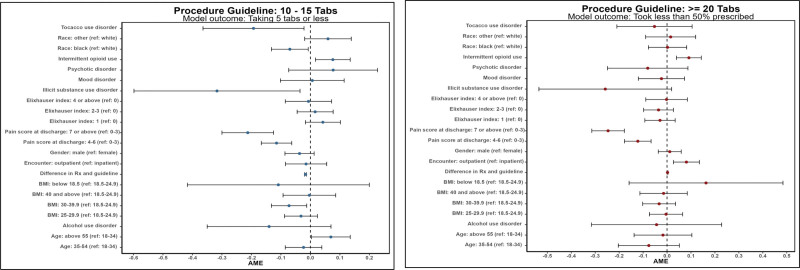
AME of patient-reported use compared against guideline recommendation. AME, average marginal effect.

We investigated patient reported use relative to the health system guideline recommendation and completed an adjusted analysis with procedures grouped by recommendation quantity being 10–15 oxycodone (5 mg) tablets or 20 or higher. In the adjusted analysis of patient reported opioid use *relative to the guideline recommend quantity*, we find higher opioid use significantly associated with older age, male sex, obese body mass index, discharge pain score, and history of mood disorder. Figure 1 displays point estimates of this analysis. Similar analyses were completed for procedures where the guideline recommendation was 20 or more. Higher pain score at discharge, older age, and history of substance use disorder were significantly associated with higher opioid use relative to prescription quantity. When comparing use to guideline recommendation specifically, only discharge pain score remained significantly associated with higher use.

**FIGURE 2.**
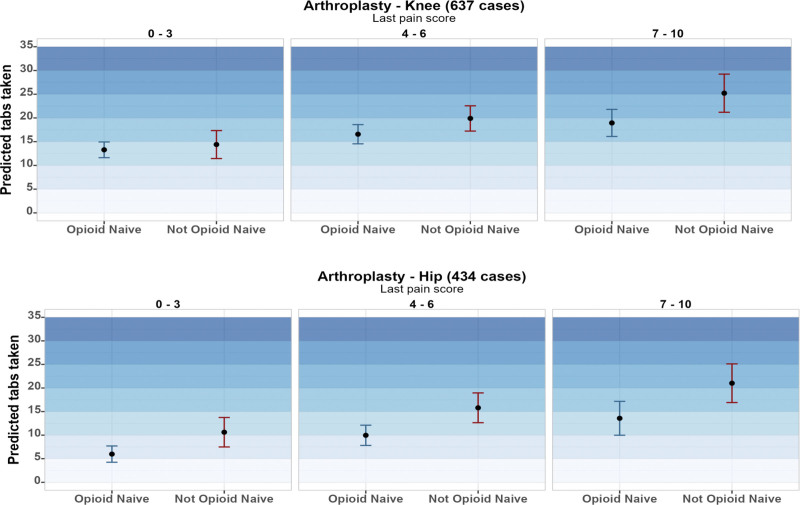
Prediction model from last pain score—opioids used for knee and hip arthroplasty.

Figure 2 reports the predicted number of opioids taken according to patient opioid naive status and last pain score prior to discharge for the 2 highest volume procedures (knee and hip arthroplasty). See supplement for this model across guideline quantities, of note over the study period across all procedures 50.2% of prescriptions were within the recommended guideline (Supplement eFigure1 http://links.lww.com/AOSO/A269). Supplement eFigure 2 http://links.lww.com/AOSO/A269 displays average marginal effect of patient reported use compared against the prescribed quantity.

### Zero to Low Opioid Use

We also investigated patient-level characteristics to determine if there were associations with individuals who had zero to low opioid use (less than a quantity of 5, 5 mg oxycodone tablets). In the adjusted model for patients undergoing procedures where the guideline amount was 15 tablets or less, we found younger age and being opioid-naïve to be associated with low to zero use. For procedures where the guideline recommendation was 20 or more tablets, we found the same significant associations. Patient-level characteristics and the odds ratio for taking zero or low quantities of opioid tablets across both groupings are provided in the supplement (Supplement eFigure3 http://links.lww.com/AOSO/A269).

## LIMITATIONS

This study has limitations. It was conducted within a single academic health care system with relatively low baseline prescribing compared with national prescribing patterns. Although incorporating data collection via an automated text messaging platform into routine care allowed us to solicit responses from all eligible patients, selection and nonresponse bias remained because individuals had to opt in for the text messaging survey (Table 1). However, response rates to the postoperative survey were higher than those for standard patient experience surveys collected as part of routine care.^26^

Participants needed to have regular access to a text message-capable device. Responding patients were more likely to be younger, male, and white. Future efforts to develop guidelines using patient-reported data would benefit from assessment of heterogeneity in patient-reported pain needs in consideration of these characteristics to account for the degree of nonresponse in various populations. Nonetheless, to our knowledge, this study is among the first to prospectively investigate patient-reported outcomes with a remote and automated method for common surgical procedures when short-term opioids are prescribed. We also conducted an analysis of opioid use relative to a guideline recommendation in attempt to account for prescribing biases which have been previously demonstrated.

In addition, because we relied on self-reporting, patients might have altered their responses; however, no clinical changes were made to an individual’s care based on responses, and participants were notified that data collection was only for research. It may also be that patients experienced improved pain scores and less need for opioids simply because they knew they were part of a research study. In addition, patients may have adjusted their use or been more conscious of their use in the context of increased media coverage and public awareness about the opioid crisis in the United States. However, even if patients adjusted their use or reported use for these reasons, there is little reason to expect that this would have influenced the associations observed between various patient characteristics and opioid use.

## DISCUSSION

The practice of advancing opioid stewardship following surgery is important to ensure that severe pain is adequately managed, and the risks associated with prescription opioids are minimized. Using an automated patient-reported data capture strategy, this study sought to investigate the associations between patient-level factors and patient-reported opioid use to determine generalizable factors that can be used by clinicians to tailor postoperative opioid prescribing. The study builds upon prior research and has 3 main findings which help to inform and guide clinicians in future pain management.

First, a distinct patient-reported measure that can help assist prescribers in anticipating opioid use following surgery may be the discharge pain score. Prior surgical research has investigated the associations between lower pain score and lower opioid use at large.^27,28^ Often upon discharge, clinicians will assess patient-reported pain prior to discharge and in our analyses, this was a significantly associated with opioid use. We found that patients in this cohort who reported their pain score at 2 (on a scale of 0–10 as commonly used) at discharge were more likely to use zero or low amounts of their opioid prescription. Interestingly, patients with a history of prior opioid exposure and higher discharge pain (scores >7) showed trends towards roughly using 5 additional tablets for high volume procedures such as knee or hip arthroplasty. These findings reveal insights toward tailoring prescribing to both discharge pain and history of opioid exposure. Additional research in addressing postsurgical pain for patients with history of opioid use is needed. Clinical decision support tools continue to expand with the EHR and help promote best practices, reduce low value care, and provide safety and quality checks. EHR nudges also have been shown to reduce opioid prescribing variation in clinical settings. Incorporating a patient’s discharge pain score into these workflows alongside prescriber education would provide clinicians an additional consideration to help tailor their prescribing to be dynamic and adaptive to patient needs.

Second, while patient-level variables, such as sex or race, may be associated with varying patient-reported opioid use, these associations need to be viewed within the context of prescribing behaviors and potential biases.^25^ When characterizing patient-reported use, previous research has consistently demonstrated the link between higher opioid prescription quantity and higher patient use.^15^ Understanding the nuances of prescribing versus use are important, and here we specifically investigated patient-level use relative to the recommended health system guideline to adjust for prescribing behavior variation. We found many patient-level associations to be muted and not statistically significant.

Third, there is an opportunity to minimize opioid prescribing for younger and opioid-naïve patients following surgery. Specifically, these data set demonstrated that 52.4% of opioid-naïve patients used zero or less than 5 tablets of oxycodone following their surgery. This finding may reflect a shift from prior research suggesting that younger patients may use higher quantities of opioids following surgery.^15^ A potential explanation for this may be related to the broader and national focus on opioids and opioid use disorder and the public may be exploring non-opioid pain management. These results also expand to include near real-time patient-reported data including pain, ability to manage pain, and medication use. Opioid misuse and new persistent opioid use have been associated with initial opioid prescriptions which may begin following an initial surgery. The opportunity to reduce these initial prescriptions for specific populations remains underexplored, and these findings highlight 2 identifiable patient-level characteristics to inform and guide prescribers ahead of surgery. As mentioned before, these factors could be incorporated into clinical decision support in the moment of prescribing using the EHR. Future work would investigate how these findings translate to other health systems and implementation science strategies designed to build dynamic prescribing methods to tailor opioid prescribing for surgical patients.

## CONCLUSION

In this study of patient-reported outcomes related to pain and opioid use following common surgeries, we identify patient-level characteristics to help inform and guide surgeons to dynamically tailor opioid prescribing to reduce the risks associated with opioids while also managing anticipated pain. Specifically, there are opportunities to provide clinical decision support and prescribe lower quantities to younger patients, those who are opioid-naïve, and have lower pain scores upon discharge.

## Supplementary Material


