# Supplementary material for: Identifying Patient Characteristics Associated With Opioid Use to Inform Surgical Pain Management
Source: Ann Surg Open. 2023 Nov 14;4(4):e355. doi: 10.1097/AS9.0000000000000355 (PMC10735081; doi:10.1097/AS9.0000000000000355)

Appendix/Supplement:
Procedure Grouping CPT Codes and Penn Medicine Opioid Guideline Amount

Eligible for Report	Department	Specialty/Division	Procedure Group	CPT	Opioid Guideline
1	NEUROSURGERY	NEUROSURGERY	ACDF	22551	0 - 30
1	NEUROSURGERY	NEUROSURGERY	ACDF	22552	0 - 30
1	NEUROSURGERY	NEUROSURGERY	ACDF	22554	0 - 30
1	NEUROSURGERY	NEUROSURGERY	Thoracolumbar Fusion	22532	0 - 40
1	NEUROSURGERY	NEUROSURGERY	Thoracolumbar Fusion	22533	0 - 40
1	NEUROSURGERY	NEUROSURGERY	Thoracolumbar Fusion	22534	0 - 40
1	NEUROSURGERY	NEUROSURGERY	Thoracolumbar Fusion	22556	0 - 40
1	NEUROSURGERY	NEUROSURGERY	Thoracolumbar Fusion	22558	0 - 40
1	NEUROSURGERY	NEUROSURGERY	Thoracolumbar Fusion	22585	0 - 40
1	NEUROSURGERY	NEUROSURGERY	Thoracolumbar Fusion	22586	0 - 40
1	NEUROSURGERY	NEUROSURGERY	Thoracolumbar Fusion	22610	0 - 40
1	NEUROSURGERY	NEUROSURGERY	Thoracolumbar Fusion	22612	0 - 40
1	NEUROSURGERY	NEUROSURGERY	Thoracolumbar Fusion	22614	0 - 40
1	NEUROSURGERY	NEUROSURGERY	Thoracolumbar Fusion	22630	0 - 40
1	NEUROSURGERY	NEUROSURGERY	Thoracolumbar Fusion	22632	0 - 40
1	NEUROSURGERY	NEUROSURGERY	Thoracolumbar Fusion	22633	0 - 40
1	NEUROSURGERY	NEUROSURGERY	Thoracolumbar Fusion	22634	0 - 40
1	NEUROSURGERY	NEUROSURGERY	Thoracolumbar Fusion	22800	0 - 40
1	NEUROSURGERY	NEUROSURGERY	Thoracolumbar Fusion	22802	0 - 40
1	NEUROSURGERY	NEUROSURGERY	Thoracolumbar Fusion	22804	0 - 40
1	NEUROSURGERY	NEUROSURGERY	Thoracolumbar Laminectomy	62380	0 - 30
1	NEUROSURGERY	NEUROSURGERY	Thoracolumbar Laminectomy	63001	0 - 30
1	NEUROSURGERY	NEUROSURGERY	Thoracolumbar Laminectomy	63005	0 - 30
1	NEUROSURGERY	NEUROSURGERY	Thoracolumbar Laminectomy	63012	0 - 30
1	NEUROSURGERY	NEUROSURGERY	Thoracolumbar Laminectomy	63015	0 - 30
1	NEUROSURGERY	NEUROSURGERY	Thoracolumbar Laminectomy	63016	0 - 30
1	NEUROSURGERY	NEUROSURGERY	Thoracolumbar Laminectomy	63017	0 - 30
1	NEUROSURGERY	NEUROSURGERY	Thoracolumbar Laminectomy	63020	0 - 30
1	NEUROSURGERY	NEUROSURGERY	Thoracolumbar Laminectomy	63030	0 - 30
1	NEUROSURGERY	NEUROSURGERY	Thoracolumbar Laminectomy	63035	0 - 30
1	NEUROSURGERY	NEUROSURGERY	Thoracolumbar Laminectomy	63040	0 - 30
1	NEUROSURGERY	NEUROSURGERY	Thoracolumbar Laminectomy	63042	0 - 30
1	NEUROSURGERY	NEUROSURGERY	Thoracolumbar Laminectomy	63043	0 - 30
1	NEUROSURGERY	NEUROSURGERY	Thoracolumbar Laminectomy	63044	0 - 30
1	NEUROSURGERY	NEUROSURGERY	Thoracolumbar Laminectomy	63045	0 - 30
1	NEUROSURGERY	NEUROSURGERY	Thoracolumbar Laminectomy	63046	0 - 30
1	NEUROSURGERY	NEUROSURGERY	Thoracolumbar Laminectomy	63047	0 - 30
1	NEUROSURGERY	NEUROSURGERY	Thoracolumbar Laminectomy	63048	0 - 30
1	NEUROSURGERY	NEUROSURGERY	Thoracolumbar Laminectomy	63055	0 - 30
1	NEUROSURGERY	NEUROSURGERY	Thoracolumbar Laminectomy	63056	0 - 30
1	NEUROSURGERY	NEUROSURGERY	Thoracolumbar Laminectomy	63057	0 - 30
1	NEUROSURGERY	NEUROSURGERY	Thoracolumbar Laminectomy	63075	0 - 30
1	NEUROSURGERY	NEUROSURGERY	Thoracolumbar Laminectomy	63076	0 - 30

1	NEUROSURGERY	NEUROSURGERY	Thoracolumbar Laminectomy	63081	0 - 30
1	NEUROSURGERY	NEUROSURGERY	Thoracolumbar Laminectomy	63082	0 - 30
1	NEUROSURGERY	NEUROSURGERY	Thoracolumbar Laminectomy	63085	0 - 30
1	NEUROSURGERY	NEUROSURGERY	Thoracolumbar Laminectomy	63086	0 - 30
1	NEUROSURGERY	NEUROSURGERY	Thoracolumbar Laminectomy	63102	0 - 30
1	NEUROSURGERY	NEUROSURGERY	Thoracolumbar Laminectomy	63103	0 - 30
1	NEUROSURGERY	NEUROSURGERY	Thoracolumbar Laminectomy	63265	0 - 30
1	NEUROSURGERY	NEUROSURGERY	Thoracolumbar Laminectomy	63266	0 - 30
1	NEUROSURGERY	NEUROSURGERY	Thoracolumbar Laminectomy	63267	0 - 30
1	NEUROSURGERY	NEUROSURGERY	Thoracolumbar Laminectomy	63268	0 - 30
1	NEUROSURGERY	NEUROSURGERY	Thoracolumbar Laminectomy	63272	0 - 30
1	NEUROSURGERY	NEUROSURGERY	Thoracolumbar Laminectomy	63273	0 - 30
1	NEUROSURGERY	NEUROSURGERY	Thoracolumbar Laminectomy	63275	0 - 30
1	NEUROSURGERY	NEUROSURGERY	Thoracolumbar Laminectomy	63276	0 - 30
1	NEUROSURGERY	NEUROSURGERY	Thoracolumbar Laminectomy	63277	0 - 30
1	NEUROSURGERY	NEUROSURGERY	Thoracolumbar Laminectomy	63280	0 - 30
1	NEUROSURGERY	NEUROSURGERY	Thoracolumbar Laminectomy	63281	0 - 30
1	NEUROSURGERY	NEUROSURGERY	Thoracolumbar Laminectomy	63282	0 - 30
1	NEUROSURGERY	NEUROSURGERY	Thoracolumbar Laminectomy	63290	0 - 30
1	NEUROSURGERY	NEUROSURGERY	Thoracolumbar Laminectomy	63295	0 - 30
1	ORTHOPAEDICS	ORTHOPAEDICS	Arthroplasty - Hip	27137	0 - 20
1	ORTHOPAEDICS	ORTHOPAEDICS	Arthroplasty - Hip	27138	0 - 20
1	ORTHOPAEDICS	ORTHOPAEDICS	Arthroplasty - Hip	27134	0 - 20
1	ORTHOPAEDICS	ORTHOPAEDICS	Arthroplasty - Hip	27132	0 - 20
1	ORTHOPAEDICS	ORTHOPAEDICS	Arthroplasty - Hip	27130	0 - 20
1	ORTHOPAEDICS	ORTHOPAEDICS	Arthroplasty - Knee	27488	0 - 25
1	ORTHOPAEDICS	ORTHOPAEDICS	Arthroplasty - Knee	27441	0 - 25
1	ORTHOPAEDICS	ORTHOPAEDICS	Arthroplasty - Knee	27442	0 - 25
1	ORTHOPAEDICS	ORTHOPAEDICS	Arthroplasty - Knee	27438	0 - 25
1	ORTHOPAEDICS	ORTHOPAEDICS	Arthroplasty - Knee	27443	0 - 25
1	ORTHOPAEDICS	ORTHOPAEDICS	Arthroplasty - Knee	27440	0 - 25
1	ORTHOPAEDICS	ORTHOPAEDICS	Arthroplasty - Knee	27420	0 - 25
1	ORTHOPAEDICS	ORTHOPAEDICS	Arthroplasty - Knee	27422	0 - 25
1	ORTHOPAEDICS	ORTHOPAEDICS	Arthroplasty - Knee	27381	0 - 25
1	ORTHOPAEDICS	ORTHOPAEDICS	Arthroplasty - Knee	27380	0 - 25
1	ORTHOPAEDICS	ORTHOPAEDICS	Arthroplasty - Knee	27418	0 - 25
1	ORTHOPAEDICS	ORTHOPAEDICS	Arthroplasty - Knee	27487	0 - 25
1	ORTHOPAEDICS	ORTHOPAEDICS	Arthroplasty - Knee	27486	0 - 25
1	ORTHOPAEDICS	ORTHOPAEDICS	Arthroplasty - Knee	27447	0 - 25
1	ORTHOPAEDICS	ORTHOPAEDICS	Arthroplasty - Knee	27446	0 - 25
1	ORTHOPAEDICS	ORTHOPAEDICS	Arthroplasty - Shoulder	23470	0 - 25
1	ORTHOPAEDICS	ORTHOPAEDICS	Arthroplasty - Shoulder	23474	0 - 25
1	ORTHOPAEDICS	ORTHOPAEDICS	Arthroplasty - Shoulder	23473	0 - 25
1	ORTHOPAEDICS	ORTHOPAEDICS	Arthroplasty - Shoulder	23472	0 - 25
1	ORTHOPAEDICS	ORTHOPAEDICS	Fracture Treatment - Ankle except trimalleolar	27818	0 - 15
1	ORTHOPAEDICS	ORTHOPAEDICS	Fracture Treatment - Ankle except trimalleolar	27814	0 - 15

1	ORTHOPAEDICS	ORTHOPAEDICS	Fracture Treatment - Ankle except trimalleolar	27810	0 - 15
1	ORTHOPAEDICS	ORTHOPAEDICS	Fracture Treatment - Ankle except trimalleolar	27792	0 - 15
1	ORTHOPAEDICS	ORTHOPAEDICS	Fracture Treatment - Ankle except trimalleolar	27784	0 - 15
1	ORTHOPAEDICS	ORTHOPAEDICS	Fracture Treatment - Ankle except trimalleolar	27766	0 - 15
1	ORTHOPAEDICS	ORTHOPAEDICS	Fracture Treatment - Radius/Ulna	25600	0 - 15
1	ORTHOPAEDICS	ORTHOPAEDICS	Fracture Treatment - Radius/Ulna	25605	0 - 15
1	ORTHOPAEDICS	ORTHOPAEDICS	Fracture Treatment - Radius/Ulna	25525	0 - 15
1	ORTHOPAEDICS	ORTHOPAEDICS	Fracture Treatment - Radius/Ulna	25608	0 - 15
1	ORTHOPAEDICS	ORTHOPAEDICS	Fracture Treatment - Radius/Ulna	25526	0 - 15
1	ORTHOPAEDICS	ORTHOPAEDICS	Fracture Treatment - Radius/Ulna	24685	0 - 15
1	ORTHOPAEDICS	ORTHOPAEDICS	Fracture Treatment - Radius/Ulna	25609	0 - 15
1	ORTHOPAEDICS	ORTHOPAEDICS	Fracture Treatment - Radius/Ulna	25520	0 - 15
1	ORTHOPAEDICS	ORTHOPAEDICS	Fracture Treatment - Radius/Ulna	25607	0 - 15
1	ORTHOPAEDICS	ORTHOPAEDICS	Fracture Treatment - Radius/Ulna	25606	0 - 15
1	ORTHOPAEDICS	ORTHOPAEDICS	Fracture Treatment - Radius/Ulna	25575	0 - 15
1	ORTHOPAEDICS	ORTHOPAEDICS	Fracture Treatment - Radius/Ulna	25574	0 - 15
1	ORTHOPAEDICS	ORTHOPAEDICS	Fracture Treatment - Radius/Ulna	25565	0 - 15
1	ORTHOPAEDICS	ORTHOPAEDICS	Fracture Treatment - Radius/Ulna	25545	0 - 15
1	ORTHOPAEDICS	ORTHOPAEDICS	Fracture Treatment - Radius/Ulna	25535	0 - 15
1	ORTHOPAEDICS	ORTHOPAEDICS	Fracture Treatment - Radius/Ulna	25515	0 - 15
1	ORTHOPAEDICS	ORTHOPAEDICS	Fracture Treatment - Radius/Ulna	25505	0 - 15
1	ORTHOPAEDICS	ORTHOPAEDICS	Fracture Treatment - Radius/Ulna	24670	0 - 15
1	ORTHOPAEDICS	ORTHOPAEDICS	Fracture Treatment - Radius/Ulna	24666	0 - 15
1	ORTHOPAEDICS	ORTHOPAEDICS	Fracture Treatment - Radius/Ulna	24665	0 - 15
1	ORTHOPAEDICS	ORTHOPAEDICS	Knee Procedures - Ligament Procedures	29888	0 - 15
1	ORTHOPAEDICS	ORTHOPAEDICS	Knee Procedures - Ligament Procedures	29889	0 - 15
1	ORTHOPAEDICS	ORTHOPAEDICS	Shoulder Procedures - Arthroscopy	29826	0 - 15
1	ORTHOPAEDICS	ORTHOPAEDICS	Shoulder Procedures - Arthroscopy	29825	0 - 15
1	ORTHOPAEDICS	ORTHOPAEDICS	Shoulder Procedures - Arthroscopy	29824	0 - 15
1	ORTHOPAEDICS	ORTHOPAEDICS	Shoulder Procedures - Arthroscopy	29823	0 - 15
1	ORTHOPAEDICS	ORTHOPAEDICS	Shoulder Procedures - Arthroscopy	29822	0 - 15
1	ORTHOPAEDICS	ORTHOPAEDICS	Shoulder Procedures - Arthroscopy	29820	0 - 15
1	ORTHOPAEDICS	ORTHOPAEDICS	Shoulder Procedures - Arthroscopy	29819	0 - 15
1	ORTHOPAEDICS	ORTHOPAEDICS	Shoulder Procedures - Arthroscopy	29807	0 - 15
1	ORTHOPAEDICS	ORTHOPAEDICS	Shoulder Procedures - Arthroscopy	29806	0 - 15
1	ORTHOPAEDICS	ORTHOPAEDICS	Shoulder Procedures - Arthroscopy	29805	0 - 15
1	ORTHOPAEDICS	ORTHOPAEDICS	Simple Knee Arthroscopy	27415	0 - 10
1	ORTHOPAEDICS	ORTHOPAEDICS	Simple Knee Arthroscopy	29879	0 - 10
1	ORTHOPAEDICS	ORTHOPAEDICS	Simple Knee Arthroscopy	27416	0 - 10
1	ORTHOPAEDICS	ORTHOPAEDICS	Simple Knee Arthroscopy	29887	0 - 10
1	ORTHOPAEDICS	ORTHOPAEDICS	Simple Knee Arthroscopy	29885	0 - 10
1	ORTHOPAEDICS	ORTHOPAEDICS	Simple Knee Arthroscopy	29877	0 - 10
1	ORTHOPAEDICS	ORTHOPAEDICS	Simple Knee Arthroscopy	29867	0 - 10
1	ORTHOPAEDICS	ORTHOPAEDICS	Simple Knee Arthroscopy	29866	0 - 10
1	ORTHOPAEDICS	ORTHOPAEDICS	Simple Knee Arthroscopy	27403	0 - 10
1	ORTHOPAEDICS	ORTHOPAEDICS	Simple Knee Arthroscopy	29880	0 - 10

1	ORTHOPAEDICS	ORTHOPAEDICS	Simple Knee Arthroscopy	29881	0 - 10
1	ORTHOPAEDICS	ORTHOPAEDICS	Simple Knee Arthroscopy	29883	0 - 10
1	ORTHOPAEDICS	ORTHOPAEDICS	Simple Knee Arthroscopy	29882	0 - 10
1	ORTHOPAEDICS	ORTHOPAEDICS	Simple Knee Arthroscopy	29884	0 - 10
1	ORTHOPAEDICS	ORTHOPAEDICS	Simple Knee Arthroscopy	29875	0 - 10
1	ORTHOPAEDICS	ORTHOPAEDICS	Simple Knee Arthroscopy	29873	0 - 10
1	ORTHOPAEDICS	ORTHOPAEDICS	Simple Knee Arthroscopy	29871	0 - 10
1	ORTHOPAEDICS	ORTHOPAEDICS	Simple Knee Arthroscopy	29870	0 - 10
1	ORTHOPAEDICS	ORTHOPAEDICS	Simple Knee Arthroscopy	29874	0 - 10
1	ORTHOPAEDICS	ORTHOPAEDICS	Trigger Finger Release	26055	0 - 5
1	SURGERY	CARDIAC SURGERY	Aortic Valve Replacement	33405	0 - 10
1	SURGERY	CARDIAC SURGERY	Aortic Valve Replacement	33412	0 - 10
1	SURGERY	CARDIAC SURGERY	Aortic Valve Replacement	33410	0 - 10
1	SURGERY	CARDIAC SURGERY	Aortic Valve Replacement	33411	0 - 10
1	SURGERY	CARDIAC SURGERY	Aortic Valve Replacement	33406	0 - 10
1	SURGERY	CARDIAC SURGERY	Coronary Artery Bypass Graft (CABG)	33516	0 - 15
1	SURGERY	CARDIAC SURGERY	Coronary Artery Bypass Graft (CABG)	33514	0 - 15
1	SURGERY	CARDIAC SURGERY	Coronary Artery Bypass Graft (CABG)	33521	0 - 15
1	SURGERY	CARDIAC SURGERY	Coronary Artery Bypass Graft (CABG)	33517	0 - 15
1	SURGERY	CARDIAC SURGERY	Coronary Artery Bypass Graft (CABG)	33534	0 - 15
1	SURGERY	CARDIAC SURGERY	Coronary Artery Bypass Graft (CABG)	33518	0 - 15
1	SURGERY	CARDIAC SURGERY	Coronary Artery Bypass Graft (CABG)	33519	0 - 15
1	SURGERY	CARDIAC SURGERY	Coronary Artery Bypass Graft (CABG)	33513	0 - 15
1	SURGERY	CARDIAC SURGERY	Coronary Artery Bypass Graft (CABG)	33535	0 - 15
1	SURGERY	CARDIAC SURGERY	Coronary Artery Bypass Graft (CABG)	33530	0 - 15
1	SURGERY	CARDIAC SURGERY	Coronary Artery Bypass Graft (CABG)	33533	0 - 15
1	SURGERY	CARDIAC SURGERY	Coronary Artery Bypass Graft (CABG)	33511	0 - 15
1	SURGERY	CARDIAC SURGERY	Coronary Artery Bypass Graft (CABG)	33512	0 - 15
1	SURGERY	CARDIAC SURGERY	Coronary Artery Bypass Graft (CABG)	33536	0 - 15
1	SURGERY	CARDIAC SURGERY	Coronary Artery Bypass Graft (CABG)	33510	0 - 15
1	SURGERY	GENERAL SURGERY	Appendectomy - Laparoscopic	44970	0 - 10
1	SURGERY	GENERAL SURGERY	Cholecystectomy - Laparoscopic	47563	0 - 10
1	SURGERY	GENERAL SURGERY	Cholecystectomy - Laparoscopic	47564	0 - 10
1	SURGERY	GENERAL SURGERY	Cholecystectomy - Laparoscopic	47562	0 - 10
1	SURGERY	GENERAL SURGERY	Colon Resection - Laparoscopic	44204	0 - 10
1	SURGERY	GENERAL SURGERY	Colon Resection - Laparoscopic	44208	0 - 10
1	SURGERY	GENERAL SURGERY	Colon Resection - Laparoscopic	44206	0 - 10
1	SURGERY	GENERAL SURGERY	Colon Resection - Laparoscopic	44205	0 - 10
1	SURGERY	GENERAL SURGERY	Colon Resection - Laparoscopic	44210	0 - 10
1	SURGERY	GENERAL SURGERY	Colon Resection - Laparoscopic	44207	0 - 10
1	SURGERY	GENERAL SURGERY	Colon Resection - Laparoscopic	44212	0 - 10
1	SURGERY	GENERAL SURGERY	Colon Resection - Laparoscopic	44211	0 - 10
1	SURGERY	GENERAL SURGERY	Gastric Restrictive Procedure - Laparoscopic	43774	0 - 10
1	SURGERY	GENERAL SURGERY	Gastric Restrictive Procedure - Laparoscopic	43772	0 - 10
1	SURGERY	GENERAL SURGERY	Gastric Restrictive Procedure - Laparoscopic	43770	0 - 10
1	SURGERY	GENERAL SURGERY	Gastric Restrictive Procedure - Laparoscopic	43775	0 - 10

1	SURGERY	GENERAL SURGERY	Inguinal Hernia Repair	49525	0 - 10
1	SURGERY	GENERAL SURGERY	Inguinal Hernia Repair	49521	0 - 10
1	SURGERY	GENERAL SURGERY	Inguinal Hernia Repair	49520	0 - 10
1	SURGERY	GENERAL SURGERY	Inguinal Hernia Repair	49507	0 - 10
1	SURGERY	GENERAL SURGERY	Inguinal Hernia Repair	49505	0 - 10
1	SURGERY	GENERAL SURGERY	Inguinal Hernia Repair	49501	0 - 10
1	SURGERY	GENERAL SURGERY	Inguinal Hernia Repair - LAPAROSCOPY	49651	0 - 5
1	SURGERY	GENERAL SURGERY	Inguinal Hernia Repair - LAPAROSCOPY	49650	0 - 5
1	SURGERY	GENERAL SURGERY	Kidney Transplant Procedures	50340	0 - 10
1	SURGERY	GENERAL SURGERY	Kidney Transplant Procedures	50547	0 - 10
1	SURGERY	GENERAL SURGERY	Kidney Transplant Procedures	50370	0 - 10
1	SURGERY	GENERAL SURGERY	Kidney Transplant Procedures	50360	0 - 10
1	SURGERY	GENERAL SURGERY	Kidney Transplant Procedures	50320	0 - 10
1	SURGERY	GENERAL SURGERY	Removal - Kidney - Laparoscopic	50548	0 - 10
1	SURGERY	GENERAL SURGERY	Removal - Kidney - Laparoscopic	50546	0 - 10
1	SURGERY	GENERAL SURGERY	Removal - Kidney - Laparoscopic	50545	0 - 10
1	SURGERY	GENERAL SURGERY	Removal - Kidney - Laparoscopic	50543	0 - 10
1	SURGERY	GENERAL SURGERY	Thyroidectomy	60271	0 - 5
1	SURGERY	GENERAL SURGERY	Thyroidectomy	60270	0 - 5
1	SURGERY	GENERAL SURGERY	Thyroidectomy	60260	0 - 5
1	SURGERY	GENERAL SURGERY	Thyroidectomy	60254	0 - 5
1	SURGERY	GENERAL SURGERY	Thyroidectomy	60252	0 - 5
1	SURGERY	GENERAL SURGERY	Thyroidectomy	60240	0 - 5
1	SURGERY	GENERAL SURGERY	Thyroidectomy	60225	0 - 5
1	SURGERY	GENERAL SURGERY	Thyroidectomy	60220	0 - 5
1	SURGERY	GENERAL SURGERY	Thyroidectomy	60212	0 - 5
1	SURGERY	GENERAL SURGERY	Thyroidectomy	60210	0 - 5
1	SURGERY	GENERAL SURGERY	Umbilical Hernia Repair	49587	0 - 15
1	SURGERY	GENERAL SURGERY	Umbilical Hernia Repair	49585	0 - 15
1	SURGERY	GENERAL SURGERY	Umbilical Hernia Repair	49582	0 - 15
1	SURGERY	GENERAL SURGERY	Umbilical Hernia Repair	49580	0 - 15
1	SURGERY	GENERAL SURGERY	Ventral Hernia Repair	49568	0 - 15
1	SURGERY	GENERAL SURGERY	Ventral Hernia Repair	49566	0 - 15
1	SURGERY	GENERAL SURGERY	Ventral Hernia Repair	49565	0 - 15
1	SURGERY	GENERAL SURGERY	Ventral Hernia Repair	49561	0 - 15
1	SURGERY	GENERAL SURGERY	Ventral Hernia Repair	49560	0 - 15
1	SURGERY	SURGICAL ONCOLOGY	Breast Reconstruction/Revision	19367	0 - 15
1	SURGERY	SURGICAL ONCOLOGY	Breast Reconstruction/Revision	19361	0 - 15
1	SURGERY	SURGICAL ONCOLOGY	Breast Reconstruction/Revision	19355	0 - 15
1	SURGERY	SURGICAL ONCOLOGY	Breast Reconstruction/Revision	19370	0 - 15
1	SURGERY	SURGICAL ONCOLOGY	Breast Reconstruction/Revision	19357	0 - 15
1	SURGERY	SURGICAL ONCOLOGY	Breast Reconstruction/Revision	19350	0 - 15
1	SURGERY	SURGICAL ONCOLOGY	Breast Reconstruction/Revision	19364	0 - 15
1	SURGERY	SURGICAL ONCOLOGY	Breast Reconstruction/Revision	19380	0 - 15
1	SURGERY	SURGICAL ONCOLOGY	Breast Reconstruction/Revision	19366	0 - 15
1	SURGERY	SURGICAL ONCOLOGY	Breast Reconstruction/Revision	19369	0 - 15

1	SURGERY	SURGICAL ONCOLOGY	Breast Reconstruction/Revision	19368	0 - 15
1	SURGERY	SURGICAL ONCOLOGY	Breast Reconstruction/Revision	19371	0 - 15
1	SURGERY	SURGICAL ONCOLOGY	Mastectomy, simple	19304	0 - 10
1	SURGERY	SURGICAL ONCOLOGY	Mastectomy, simple	19303	0 - 10
1	SURGERY	THORACIC SURGERY	Lung Removal	32670	0 - 15
1	SURGERY	THORACIC SURGERY	Lung Removal	32671	0 - 15
1	SURGERY	THORACIC SURGERY	Lung Removal	32669	0 - 15
1	SURGERY	THORACIC SURGERY	Lung Removal	32663	0 - 15
1	SURGERY	THORACIC SURGERY	Lung Removal	32667	0 - 15
1	SURGERY	THORACIC SURGERY	Lung Removal	32668	0 - 15
1	SURGERY	THORACIC SURGERY	Lung Removal	32666	0 - 15
1	SURGERY	UROLOGY	Radical Prostatectomy	38571	0 - 5
1	SURGERY	UROLOGY	Radical Prostatectomy	55866	0 - 5
1	SURGERY	UROLOGY	Radical Prostatectomy	55845	0 - 5
1	SURGERY	UROLOGY	Radical Prostatectomy	55831	0 - 5
1	SURGERY	UROLOGY	Radical Prostatectomy	55821	0 - 5
1	SURGERY	UROLOGY	Vasectomy	55250	0 - 5

eFigure1: Prediction Model Including Discharge Pain Score, Opioid Exposure, and Guideline Recommendations

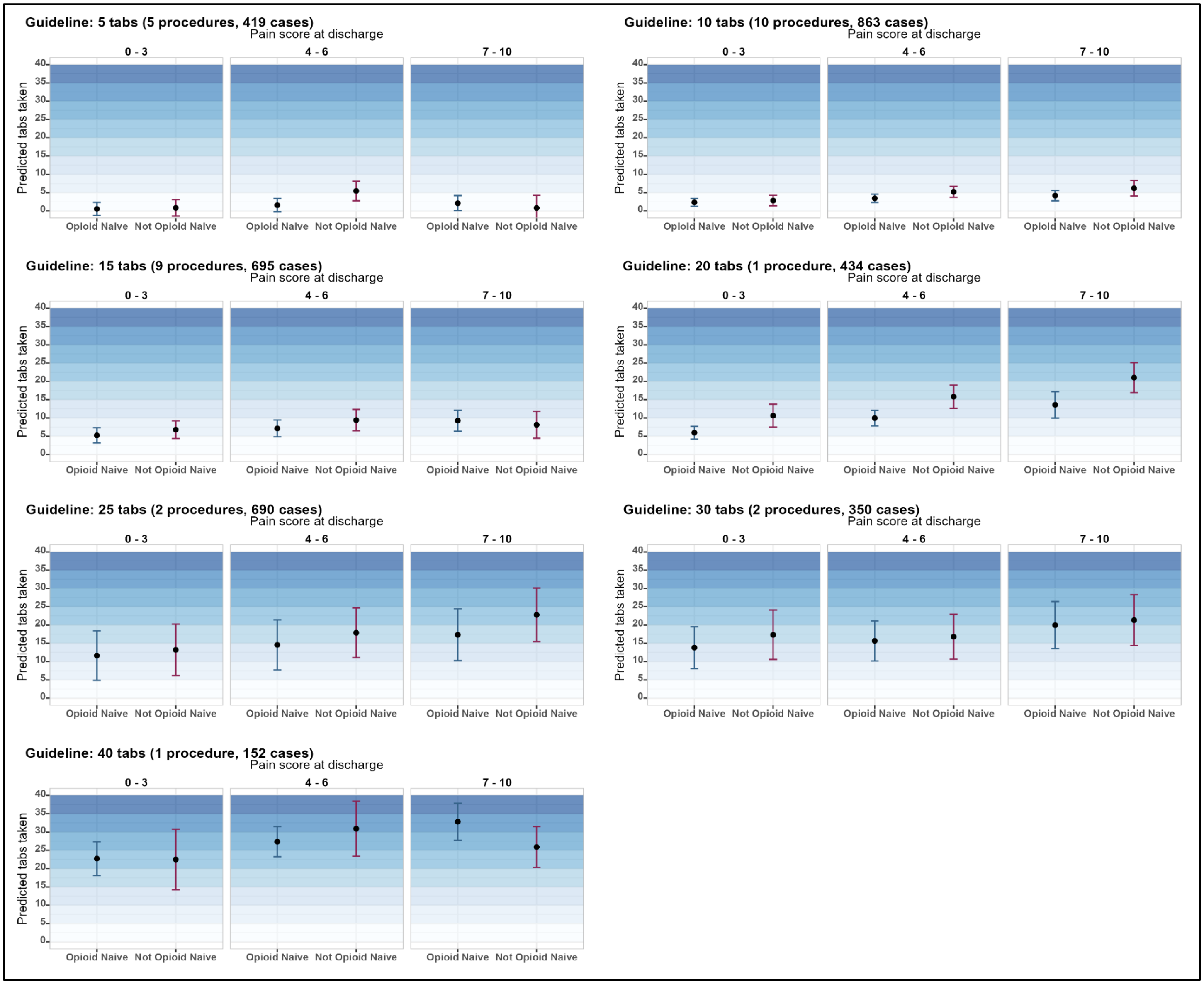

eFigure 2: Average Marginal Effect (AME) of Patient Reported Use Compared Against Prescribed Quantity

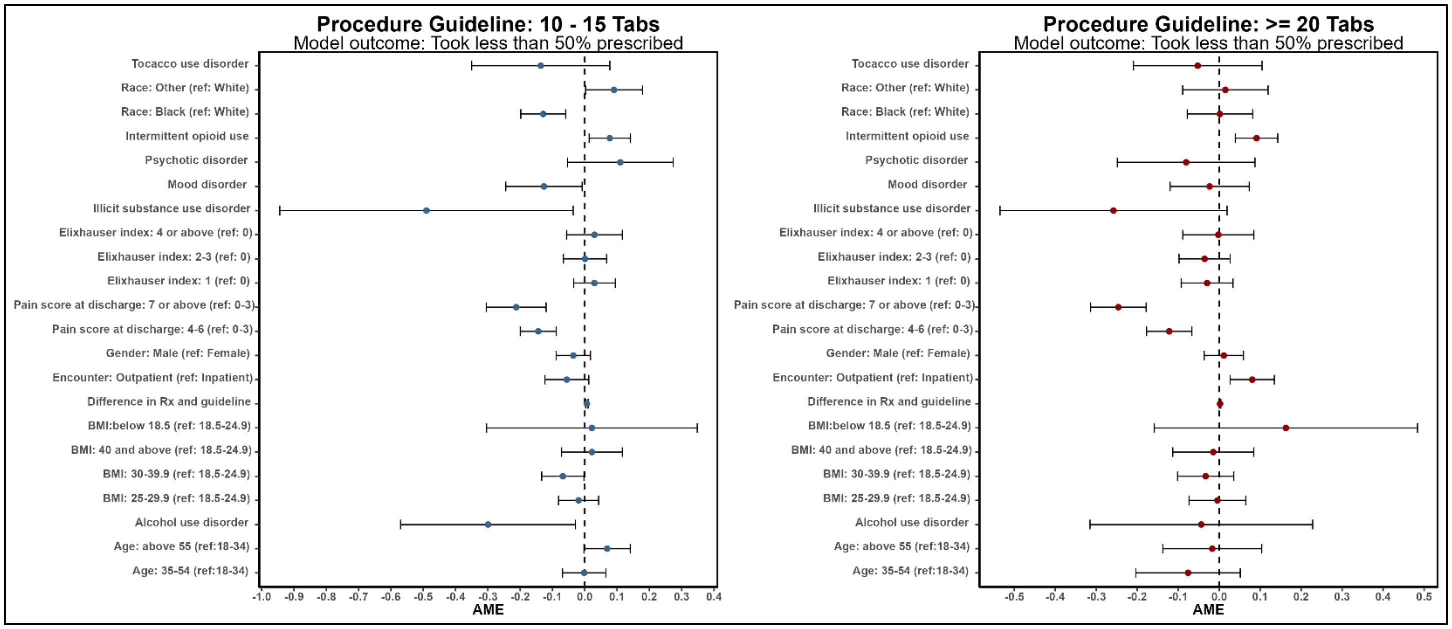

eFigure 3: Average Marginal Effect of Patient Reported Low or No Use

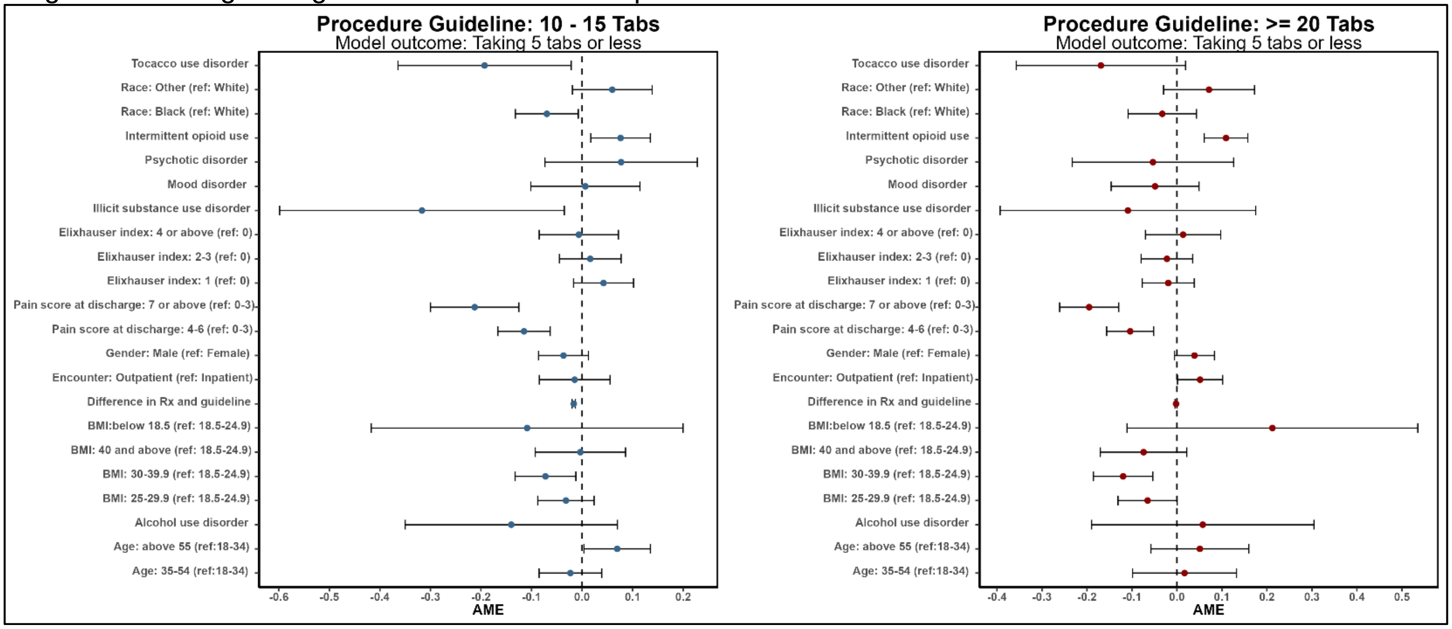

Supplement: Supplementary file 1 [file as9-4-e355-s001.pdf]
